# Purification and identification of an antioxidant peptide from perilla seed (*Perilla frutescens*) meal protein hydrolysate

**DOI:** 10.1002/fsn3.998

**Published:** 2019-04-23

**Authors:** Ja Min Kim, Andrea M. Liceaga, Kyung Young Yoon

**Affiliations:** ^1^ Department of Food and Nutrition Yeungnam University Gyeongsan Korea; ^2^ Department of Food Science Purdue University West Lafayette Indiana

**Keywords:** antioxidant peptides, perilla seed meal, purification

## Abstract

This study aimed to obtain antioxidant peptides from perilla seed meal (PSM), which is normally discarded as an industrial waste during seed oil extraction. PSM protein was hydrolyzed using trypsin and fractionated by ultrafiltration. Molecular weight fraction (<3 kDa) with the highest antioxidant activity was purified using prep‐HPLC and analytical HPLC. The purification fold of the peptide (fraction V) obtained from PSM protein hydrolysate on DPPH radical scavenging activity, ABTS radical scavenging activity, and reducing power was 1.79‐, 1.59‐, and 1.81‐fold, respectively, after the three‐step purification procedure. The sequence of the purified peptide from fraction V that exhibited free radical scavenging activity and reducing power was identified as Ile‐Ser‐Pro‐Arg‐Ile‐Leu‐Ser‐Tyr‐Asn‐Leu‐Arg (1,330.77 Da). These results demonstrate that PSM protein, a by‐product from the oil seed extraction, can be used as a source of natural antioxidant peptides for food and/or nutraceutical applications.

## 1 INTRODUCTION

Free radicals can affect diverse functions, more essentially the energy supply, detoxification, chemical‐signaling, and defense against infections (Jiang et al., 2014; Kim, Je, & Kim, 2007; Shahidi & Zhong, 2010). However, the excessive amounts of free radicals are associated with several diseases, including atherosclerosis, cancer, diabetes mellitus, and neurodegenerative disease, among others (Butterfield et al., 2002; Kim & Wijesekara, 2010). In addition, oxidation in foods causes food quality deterioration, such as inducing rancid aromas and flavors, and decreasing the product's shelf life (Kim & Wijesekara, 2010). Therefore, natural and synthetic antioxidants have commonly been used for radical scavenging in biological systems. Although synthetic antioxidants are effective and inexpensive; however, their use is strictly regulated owing to their toxicity and side effects on human health (Ito et al., 1986; Shahidi & Zhong, 2010). In the past few years, new interest has emerged to find natural antioxidants, such as carotenoids, flavonoids, phenolic compounds, and peptides (Memarpoor‐Yazdi, Asoodeh, & Chamani, 2012; You, Udenigwe, Aluko, & Wu, 2010). Among these, the naturally derived antioxidant peptides have some advantages compared to synthetic compounds owing to their simpler structure and lower molecular weight, higher stability under different conditions, and no hazardous immunoreactions (Sarmadi & Ismail, 2010). For these reasons, scientists are looking for antioxidant peptides from various natural sources, such as soybean (Chen, Muramoto, Yamauchi, & Nokihara, 1996), oilseed (Rhee, Ziprin, & Rhee, 1979), giant squid muscle (Rajapakse, Mendis, Byun, & Kim, 2005), sardinella (Bougatef et al., 2010), sandfish (Jang, Liceaga, & Yoon, 2016), tuna dark muscle by‐product (Hsu, 2010), fish frame protein hydrolysates (Ketnawa & Liceaga, 2017), and fish skin gelatin (Mendis, Rajapakse, & Kim, 2005). Natural‐derived antioxidant peptides are 2–20 amino acid residues long, and their activities are dependent on their sequence, structure, and hydrophobicity (Rahman et al., 2018). The antioxidant peptides from animal sources have been well studied. Recently, interest has been emerging to purify and identify antioxidant peptides from plant sources (Jin, Liu, Zheng, Wang, & He, 2016; Zhang et al., 2018).

Perilla (*Perilla frutescens* var. *japonica* HARA), an annual plant, is cultivated throughout Asian countries. Its Korean name is “delkkae” and its leaves are widely used as a spice, garnishment, and food colorant (Ha et al., 2012). Perilla seeds are commonly subjected to oil extraction because of the presence of enriched oil content containing a high percentage of unsaturated fatty acids (approximately more than 90%) and α‐linolenic acid (ranging from 52.58% to 61.97%; Ding, Hu, Shi, Chao, & Liu, 2012). Perilla seed meal (PSM), the residue from the seed oil extraction process, can be a potentially abundant source of structurally diverse bioactive compounds (Luther et al., 2007; Lutterodt, Slavin, Whent, Turner, & Yu, 2011). PSM contains relatively higher protein content compared to perilla seeds before oil extraction. In particular, cold‐pressed PSM may provide better health benefits because cold‐press extraction involves no heat treatment or solvents (Parry & Yu, 2004; Yu, Haley, Perret, & Harris, 2002). Therefore, owing to its high protein content, cold‐pressed PSM can be used as starting material for production of antioxidant peptides (Di Bernardini et al., 2011). Although PSM is rich in protein, most of the PSM is discarded as waste or used for animal feed owing to its low cost. Therefore, in this study, PSM was hydrolyzed to develop novel antioxidant peptides, as a way of better utilizing this valuable resource.

## 2 MATERIALS AND METHODS

### 2.1 Materials

Perilla seed meal was procured from Sunsumi‐oil (Namyangju, Korea). It was ground and kept frozen at −42°C prior to use. Trypsin (Novobeta 115) used for hydrolysis was purchased from Novo Nordisk (Bagsvaerd, Denmark). 2, 2'‐Azino‐bis‐(3‐ethylbenzothiazoline‐6‐sulfonic acid) (ABTS), 1, 1‐diphenyl‐2‐picryl hydrazyl (DPPH), and bovine serum albumin (BSA) were purchased from Sigma‐Aldrich (St Louis, MO, USA). All other analytical grade reagents were purchased from Burdick and Jackson (Muskegon, MI, USA).

### 2.2 Preparation of protein extract from defatted PSM

Perilla seed meal protein extraction was carried out following a modified procedure by He, Girgih, Malomo, Ju, and Aluko (2013). PSM was dissolved in deionized water (1:10, w/v) and the slurry stirred for 1 hr at 25°C. After adjusting to pH 10 with 1 M NaOH, the slurry was stirred again for 1 hr at 25°C and centrifuged at 1,600 *g* for 30 min. The supernatant was collected, adjusted to pH 4.0 using 1 M HCl, and left for 30 min to allow protein precipitation. Subsequently, the mixture was centrifuged (1,600 *g*, 4°C) for 30 min. The resultant precipitate was re‐dispersed in deionized water, adjusted to pH 7.0 with 1 M NaOH, freeze dried, and stored at −20°C. This powder was subsequently referred as the PSM protein extract.

### 2.3 Preparation of hydrolysates from PSM protein

Preliminary experiments using various enzymes (alcalase, neutrase, trypsin, papain, and pepsin) showed that the most potent antioxidant activity was observed for PSM protein hydrolysate derived from a 3 hr hydrolysis using trypsin (25 units). These hydrolysis parameters were thus used in the present study. PSM protein powder was mixed with 0.1 M sodium phosphate buffer (pH 8.0) in the ratio of 1:20 (w/v), and 25 units of trypsin were added to the reaction. Hydrolysis was carried out for 3 hr at 37°C using a water bath with stirring. Hydrolysis was terminated by heating the mixture at 100°C for 10 min, followed by centrifugation at 1,600 *g* for 3 hr. The supernatant (PSM protein hydrolysate) was collected, lyophilized, and stored at −40°C until analysis.

### 2.4 Determination of degree of hydrolysis

The degree of hydrolysis (DH) was estimated according to Jang et al. (2016) based on a modification of Hoyle and Merritt's (1994) method. Briefly, PSM protein hydrolysates were mixed with 20% trichloroacetic acid (TCA; 1:1 ratio v/v) and centrifuged at 1,600 *g* for 30 min at 4°C. The soluble protein content in the supernatant was measured using the microplate bicinchoninic acid (BCA) colorimetric method described by Smith et al. (1985). Twenty microliters of each sample was added to 160 µl of BCA reagent and incubated at 37°C for 30 min. Absorbance was measured at 560 nm using a microplate spectrophotometer (Epoch, Biotek, Winooski, VT, USA). Bovine serum albumin (BSA) was used as the standard. The DH was expressed as following equation:

$$\%\;\text{DH}=\frac{10\%\text{TCA}-\text{soluble}\;\text{protein}}{\text{Total}\;\text{protein}}\;\times \;100.$$

### 2.5 Antioxidant activity determination

#### 2.5.1 DPPH radical scavenging activity

The methodology to measure DPPH radical scavenging activity was adopted from Jang et al. (2016) with modifications for using a 96‐well clear bottom microplate. Sample (100 µl) was added to 50 µl of 0.2 mM DPPH solution. After agitation for 5 s, the mixture was left at 37°C for 30 min, and the absorbance of the control or blank (*A*_control_) and sample (*A*_sample_) were measured with the help of a microplate spectrophotometer (Epoch, Biotek) at 517 nm. The scavenging activity was determined using the following equation:

$$\%\;\text{DPPH}\;\text{radical}\;\text{scavenging}\;\text{activity}=\frac{A_{\text{control}}-A_{\text{sample}}}{A_{\text{control}}}\times \;100.$$

### 2.6 ABTS radical scavenging activity

ABTS radical scavenging activity was measured according to Oh and Yoon (2018) with modifications. The ABTS radical scavenging activity reaction was an aqueous solution of 7 mM ABTS with 2.45 mM potassium persulfate, stored in the dark at room temperature for 12 hr. Before using, the solution was diluted with 80% ethanol to an absorbance at 734 nm of 0.60 ± 0.02. Then, 50 µl of diluted ABTS radical solution was mixed with 50 µl of sample or control and kept in the dark for 6 min. Absorbance values of the control (*A*_control_) and sample (*A*_sample_) were measured at 734 nm using a microplate spectrophotometer (Epoch, Biotek). ABTS scavenging activity was expressed by following equation:

$$\%\;\text{ABTS}\;\text{radical}\;\text{scavenging}\;\text{activity}=\frac{A_{\text{control}}-A_{\text{sample}}}{A_{\text{control}}}\;\times \;100.$$

### 2.7 Reducing power

The reducing power was determined following the methodology given by Yen and Chen (1995), with modifications. Sample (100 µl) was mixed with 100 µl of 0.2 M sodium phosphate buffer (pH 6.6) and 100 µl of 1% potassium ferricyanide. The solution was incubated at 50°C for 20 min. After incubation, 100 µl of 10% TCA was added. This mixture was centrifuged at 18 *g* for 10 min at 4°C. Next, 100 µl of the upper layer was mixed with 100 µl of distilled water and 200 µl of 0.1% ferric chloride, and allowed to stand at room temperature for 10 min. The solution (200 µl) was transferred to a clear bottom 96‐well microplate, and absorbance at 700 nm was measured using a microplate spectrophotometer (Epoch, Biotek).

### 2.8 Purification of antioxidant peptides

#### 2.8.1 Ultrafiltration

The tryptic PSM protein hydrolysate was fractioned according to the procedure described by Jang et al. (2016) using an Amicon stirred ultrafiltration cell (8050; Millipore, Bedford, MA, USA) with three different molecular weight cut‐off (MWCO) membranes (3, 5, and 10 kDa). Ultrafiltration was performed sequentially under nitrogen pressure. Briefly, the PSM protein hydrolysate was first filtered through a 3 kDa MWCO membrane and the filtrate was used as a <3 kDa fraction; the retentate was passed through a 5 kDa MWCO membrane to obtain a 3–5 kDa fraction. The 5 kDa retentate was then passed through a 10 kDa MWCO membrane, obtaining the 5–10 kDa fractions. The remaining filtrate after 10 kDa MWCO membrane was used as the >10 kDa fraction. The resultant four fractions were freeze dried for the determination of antioxidant activities and molecular weight distribution. Molecular weight distribution was expressed as the relative percentage of each yield.

#### 2.8.2 Preparative high‐performance liquid chromatography

The ultrafiltration fraction with the highest antioxidant activity was separated according to Jang et al. (2016), using a HPLC system equipped with an XBridge OST C_18_ preparative column (5 µm, 10 mm × 250 mm, Waters 2695; Waters Co., Milford, MA, USA). Solvents, 0.1% (v/v) trifluoroacetic acid (TFA) in distilled water (solvent A) and 0.1% TFA (v/v) in 80% acetonitrile (solvent B), were eluted at a flow rate of 5 ml/min using the following procedure: 0 min, 100% eluent A; 0–35 min, 75% eluent A; 45 min, 75% eluent A; and 70 min, 50% eluent A. The loaded fraction was divided into seven subfractions using an automatic fraction collector (Teledyne Isco, Lincoln, NE, USA). The antioxidant activities of each of the fractions were measured. The fractions exhibiting highest antioxidant activities were pooled and freeze dried.

#### 2.8.3 Analytical high‐performance liquid chromatography

Fractions were collected using a preparative high‐performance liquid chromatography (prep‐HPLC) and loaded onto an Atlantis™ dC_18_ column (5 µm, 4.6 mm × 150 mm, Waters Co.) connected to the HPLC system (Waters 2695; Waters Co.) to confirm singular peaks as described by Jang et al. (2016). Briefly, solvent A and solvent B were prepared as described in the previous section. The flow rate was 0.8 ml/min with a linear gradient of 0%–50% solvent B in 8 min. Antioxidant activity of only single peaks of the fraction was measured.

#### 2.8.4 Molecular mass distribution and amino acid sequences of purified peptides

The molecular mass distribution and amino acid sequence of the purified peptides were determined by Q‐TOF mass spectrometer (AB Sciex Triple TOF 5600+, Foster City, CA, USA) with a Thermo UHPLC Ultimate 3000 system through an electrospray ionization source (Thermo Fisher Scientific, Waltham, MA, USA). Solution A (0.1% formic acid in distilled water) and solution B (0.1% formic acid in acetonitrile) were used in the elution and loaded onto an ACQUITY UPLC BEH 130 C_18_column (1.7 µm, 2.1 mm × 50 mm, Waters Co.). The flow rate was set to 0.3 ml/min using the following conditions: 0–3 min, 99% eluent A; 3–70 min, 50% eluent A; 70–75 min, 0% eluent A; 75–80 min, 0% eluent A; 80–81 min, 99% eluent A; and 81–90 min, 99% eluent A. All experiments were carried out using positive mode with a capillary voltage of 3,500 V. The data were gathered in the centroid mode covering the mass/charge range of 100–2,500 m/z. The MS/MS spectra were examined using Protein Pilot (AB Sciex) software against the Uniprot sequence database (http://www.uniprot.org).

### 2.9 Statistical analysis

All the experiments were conducted in triplicate with results expressed as mean ± standard deviation (mean ± *SD*), unless otherwise indicated. A one‐way analysis of variance (ANOVA) was applied using the statistical software SPSS ver. 23.0 (SPSS Inc., Chicago, IL, USA), and the Duncan's multiple range test comparisons at *p* < 0.05 was run to determine significant differences.

## 3 RESULTS AND DISCUSSION

### 3.1 Degree of hydrolysis

The DH estimates the extent of hydrolysis by determining the number (%) of peptide bond cleaved, where higher enzyme concentration and hydrolysis time will lead to increased DH or smaller molecular weight peptides (Mueller & Liceaga, 2016). After hydrolyzing PSM with trypsin at 37°C for 3 hr, the DH of the PSM protein hydrolysate was 35.84% (data not shown). The DH is usually used as indicator between different protein hydrolysates because it can determine the effect it will have on the functional properties of the peptides. For example, when the DH increases, solubility of hydrolysates will increase, whereas emulsion activity index, emulsion stability index, foaming capacity, and foam stability of hydrolysates will decrease. In contrast, higher DH will result in smaller peptides that can have biological activity such as antioxidant capacity (Jang & Lee, 2005; Klompong, Benjakul, Kantachote, & Shahidi, 2007; Nguyen, Jones, Kim, San Martin‐Gonzalez, & Liceaga, 2017).

### 3.2 Purification of antioxidant peptides from PSM hydrolysate

#### 3.2.1 PSM hydrolysate fractionation by ultrafiltration

**Figure 1:**
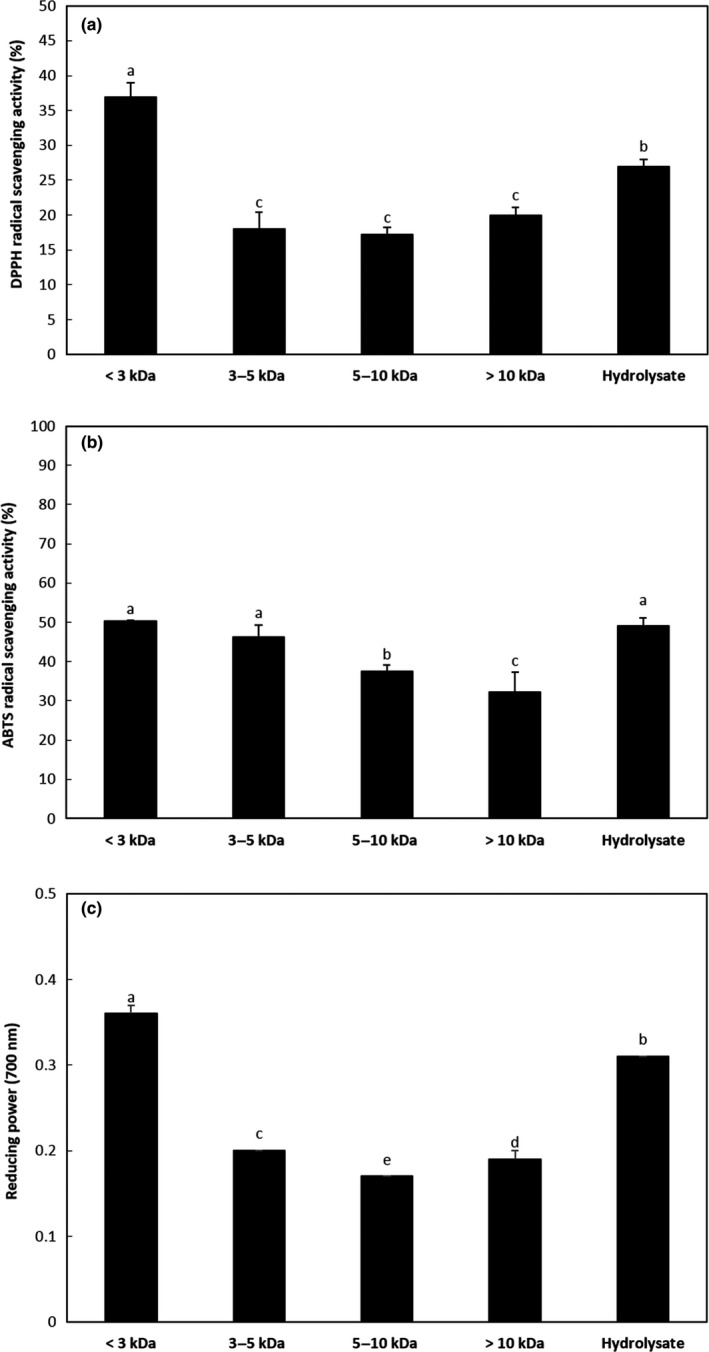
Antioxidant activities of perilla seed meal (PSM) protein hydrolysate and fractions separated by UF. (a) DPPH radical scavenging activity; (b) ABTS radical scavenging activity; (c) reducing power. DPPH radical scavenging activity and ABTS radical scavenging activity were tested at a concentration of 0.1 mg/ml. Reducing power was tested at a concentration of 1.0 mg/ml. The results are expressed as the mean ± *SD* of triplicates. Values with different letters are significantly different (*p* < 0.05)

In this study, ultrafiltration using three different MWCO membranes (10, 5, and 3 kDa) was employed to separate the PSM hydrolysate into four molecular size fractions (>10 kDa, 5–10 kDa, 3–5 kDa, and <3 kDa). The degree of molecular weight distribution is shown in Table 1. Among the four fractions, the hydrolysate with molecular weight <3 kDa accounted for 66.65% of the total hydrolysate. This result indicates that PSM hydrolysate with 35.84% DH mostly consists of low molecular weight peptides. Similar results by Zhang, Mu, and Sun (2014) showed that sweet potato hydrolysate prepared by alcalase (4% w/w) for 2 hr had the lowest molecular weight (<3 kDa) and showed highest antioxidant activity relative to the higher molecular weight fractions. It is known that peptides will have improved antioxidant activities than native proteins, mostly due to an increased availability of the functional side chain to the reactive species, and the electron‐dense peptide bonds generated by enzymatic hydrolysis (Udenigwe & Aluko, 2011). In our study, the resultant ultrafiltrates were tested for antioxidant activity. Figure 1 shows the antioxidant activities of ultrafiltered fractions and native PSM protein hydrolysate. As shown in Figure 1a, DPPH radical scavenging activities of peptide fractions (at 0.1 mg/ml) with molecular weight <3 kDa was estimated to be 36.89%, significantly higher (*p* < 0.05) than the PSM hydrolysate without ultrafiltration. In contrast, the DPPH radical scavenging activities of fractions with a molecular weight of 3–5 kDa, 5–10 kDa, and >10 kDa were significantly lower than the PSM hydrolysate. These results are in agreement with those by Li, Jiang, Zhang, Mu, and Liu (2008), where peptides from chickpea protein hydrolysate with the lowest molecular size fraction had higher DPPH radical scavenging activity. Zhuang, Zhao, and Li (2009) also observed that lower molecular weight fractions derived from jellyfish collagen hydrolysates showed the highest DPPH radical scavenging activity. Furthermore, Jang et al. (2016) reported that fractions below 3 kDa derived from sandfish hydrolysate had greater DPPH radical scavenging activity. It is proposed that the low molecular size fraction was made up of more hydrophobic amino acids capable of interacting with peroxyl DPPH radicals, as compared to the larger peptides. Moreover, DPPH radical scavenging activity by low molecular weight fractions can also be influenced by the peptides' increased solubility, allowing it to easily bind to the free radical compared to larger, less soluble peptides (Nguyen et al., 2017; Ranathunga, Rajapakse, & Kim, 2006).

**Table 1:** Molecular weight distribution of perilla seed meal (PSM) protein hydrolysates

Molecular weights (kDa)	Distribution (%)^a^
<3	66.65 ± 2.88^a^
3–5	8.21 ± 1.44^c^
5–10	6.85 ± 1.58^c^
>10	18.31 ± 0.42^b^

a Each value represents the mean ± *SD* of triplicates.

Values in the column with different superscript letters are significantly different at *p < *0.05.

Figure 1b shows the activity of original PSM protein hydrolysate and membrane fractions to scavenge the ABTS radical. Although the ABTS radical scavenging activity was observed in all fractions, the smaller fraction showed higher ABTS radical scavenging activity than the larger fraction. Therefore, the fraction with <3 kDa exhibited the highest potency, which was 50.4% at a concentration of 0.1 mg/ml. Ketnawa, Wickramathilaka, and Liceaga (2018) reported a similar trend, where fish frame protein hydrolysates with peptides (<2 kDa) exhibited the highest ABTS radical scavenging ability. In addition, hydrolysates with peptides below 10 kDa derived from corn gluten meal exhibited the highest ABTS scavenging activity (Zhuang, Tang, & Yuan, 2013). Figure 1c shows the reducing power of original PSM hydrolysate and its membrane fractions, where the low molecular weight fraction (<3 kDa) exhibited the strongest reducing power, while high molecular weight fractions possessed weaker reducing power compared to the original hydrolysate at a concentration 1 mg/ml. Low molecular weight antioxidant peptides have been widely reported in literature. As previously discussed, peptides with lower molecular weight had higher antioxidant activity (Guo, Kouzuma, & Yonekura, 2009; Jang et al., 2016; Ketnawa et al., 2018; Pownall, Udenigwe, & Aluko, 2010). The strong antioxidant properties of these peptides have been attributed to their low molecular weight, as this enhances easy reduction of radical‐mediated lipid peroxidation and the ability to react with lipid radicals (Ranathunga et al., 2006). Consequently, the < 3 kDa fraction, which showed the highest antioxidant activities, was chosen and purified for further studies.

#### 3.2.2 Purification of antioxidant peptides

**Figure 2:**
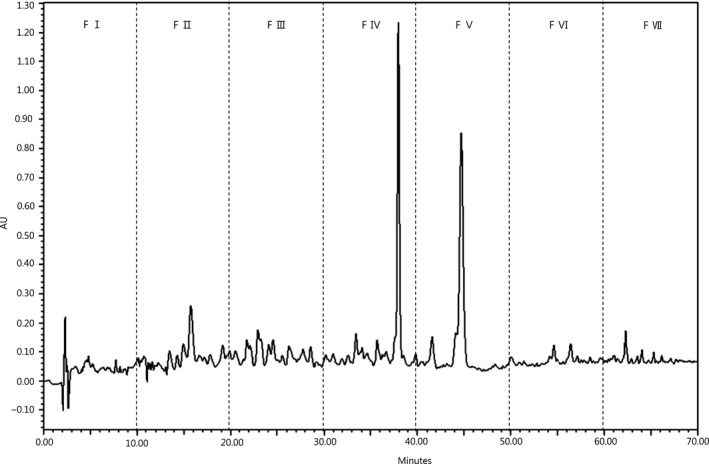
Elution profile of perilla seed meal (PSM) protein hydrolysate separated by prep‐HPLC

**Figure 3:**
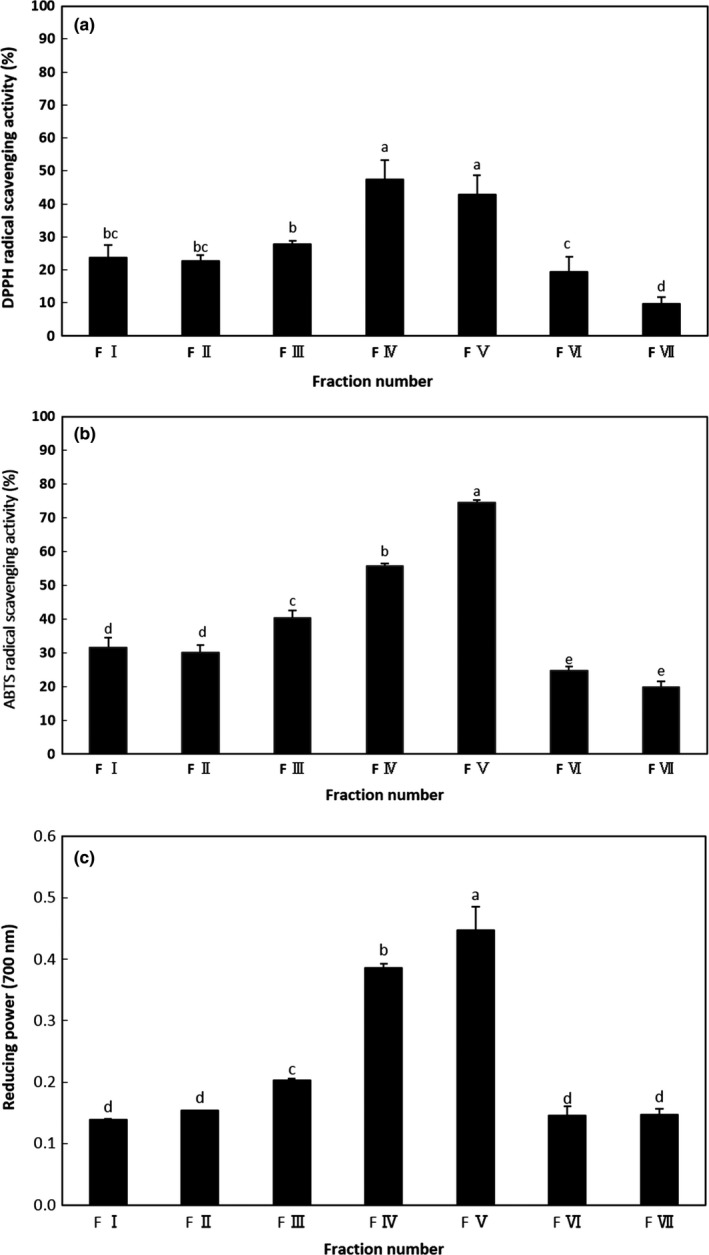
Antioxidant activities of fractions perilla seed meal (PSM) fractions separated by prep‐HPLC. (a) DPPH radical scavenging activity of the fractions separated by prep‐HPLC; (b) ABTS radical scavenging activity of the fractions separated by prep‐HPLC; (c) reducing power of the fractions separated by prep‐HPLC. DPPH radical scavenging activity and ABTS radical scavenging activity were tested at a concentration of 0.1 mg/ml. Reducing power was tested at a concentration of 1.0 mg/ml. The results are expressed as the mean ± *SD* of triplicates. Values with different letters are significantly different (*p* < 0.05)

Figure 2 shows the column chromatographic profile of the <3 kDa fraction that gave the maximum antioxidant activity, where three major peaks were eluted. The DPPH radical scavenging activities of fractions separated by prep‐HPLC are presented in Figure 3a. All the fractions displayed DPPH radical scavenging activity (at 0.1 mg/ml) with the highest activities observed in fractions IV (47.4%) and V (42.7%), whereas fractions I, II, III, VI, and VII were significantly lower (*p* < 0.05). As depicted in Figure 3b, a difference in ABTS radical scavenging was observed among the seven fractions (at 0.1 mg/ml), with fraction V being the highest (74.6%) followed by fraction IV (55.8%), with the lowest activity seen in fraction VII (19.7%). In terms of reducing power (Figure 3c), all fractions (at 1 mg/ml) showed some degree of reducing power. Higher absorbance at 700 nm indicated higher reducing power. Fraction V continued to have the highest reducing power with an absorbance of 0.45 nm, followed by fraction IV (0.39 nm). From these results, fractions IV and V showed relatively stronger antioxidant activity among the seven fractions tested.

**Figure 4:**
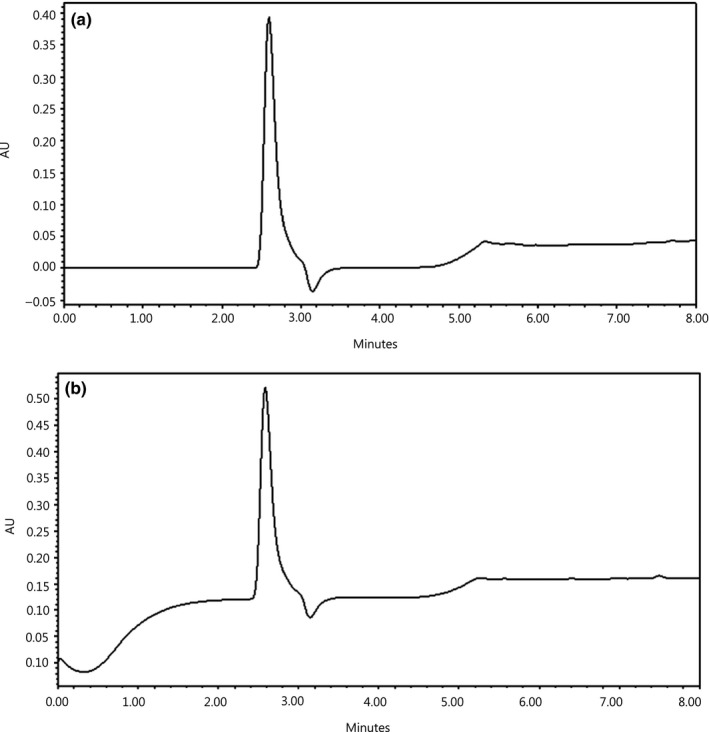
Elution profile of perilla seed meal (PSM) protein hydrolysate separated by analytical HPLC. (a) Elution profile of fraction IV separated by analytical HPLC; (b) elution profile of fraction V separated by analytical HPLC

To further purify the antioxidant peptides from these fractions (IV and V), they were loaded on a dC_18_ column using analytical HPLC. The elution profile of the peptides is given in Figure 4, demonstrating that the fractionated peptides were relatively pure. As shown in Table 2, the purification fold of the purified antioxidant peptide (PAP1) derived from fraction IV on DPPH radical scavenging activity, ABTS radical scavenging activity, and reducing power was 2.18‐, 1.38‐, and 1.40‐fold throughout the three‐step purification procedure, respectively. The purification fold of the purified antioxidant peptide (PAP2) derived from fraction V on DPPH radical scavenging activity, ABTS radical scavenging activity, and reducing power was 1.79‐, 1.59‐, and 1.81‐fold, respectively, after the three‐step purification procedure.

**Table 2:** Summary of purification of antioxidant peptides obtained from perilla seed meal (PSM) protein hydrolysate

Purification step^a^	DPPH radical scavenging activity (%)^b^	Purification fold	ABTS radical scavenging activity (%)^b^	Purification fold	Reducing power (Abs.700 nm)^b^	Purification fold	Yield (%)
Protein hydrolysate	26.93	1.00	49.12	1.00	0.31	1.00	100.00
Ultrafiltration (<3 kDa)	36.89	1.37	50.40	1.03	0.36	1.16	72.16
Prep‐HPLC							
Fraction Ⅳ	47.36	1.76	55.75	1.13	0.39	1.25	63.25
Fraction Ⅴ	39.23	1.46	74.57	1.52	0.45	1.44	54.86
Analytical HPLC							
PAP1	58.80	2.18	67.84	1.38	0.43	1.40	48.70
PAP2	48.13	1.79	78.14	1.59	0.56	1.81	46.29

Notes

a PAP1, purified antioxidant peptide derived from fraction IV; PAP2, purified antioxidant peptide derived from fraction V.

Purification fold = antioxidant activity of each fraction/antioxidant activity of PSM protein hydrolysate.

b DPPH radical scavenging and ABTS radical scavenging activities were tested at a concentration of 0.1 mg/ml. Reducing power was tested at a concentration of 1.0 mg/ml.

#### 3.2.3 Molecular mass distribution and amino acid sequences of purified peptides

**Figure 5:**
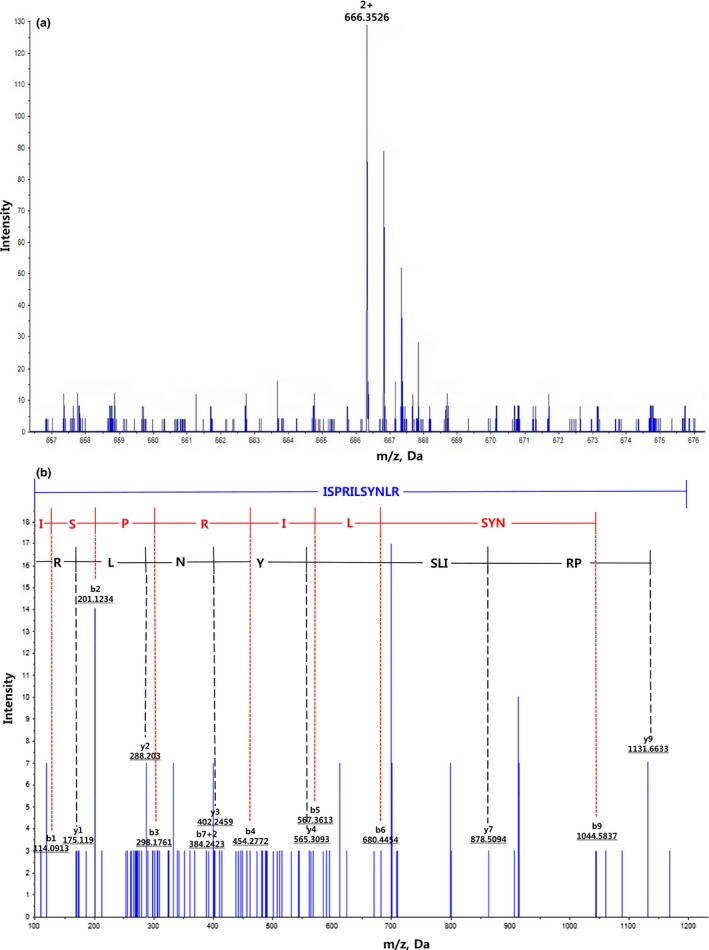
Identification of the molecular mass and amino acid sequence of purified antioxidant peptide derived from fraction V (PAP2). (a) MS spectrum of PAP2, (b) MS/MS spectrum of PAP2 and the interpretation of the obtained spectrum

The purified peptides were analyzed by Q‐TOF mass spectroscopy for identification of peptides and molecular mass distribution. The amino acid sequence of PAP1 could not be verified. This was probably because of the incorporation of unexpected interfering substances such as sulfate and phosphate during the purification or identification stage. Therefore, detailed analysis of amino acid composition and structural characteristics of PAP1 will be necessary through additional analysis. The exact MS data of PAP2 were analyzed and obtained as the molecular ion peak at m/z 666.35 [M + 2H]+, which was composed of eleven amino acids, Ile‐Ser‐Pro‐Arg‐Ile‐Leu‐Ser‐Tyr‐Asn‐Leu‐Arg, and had a molecular weight of 1,330.77 Da (Figure 5). As reported previously, shorter peptides (5–16 amino acids) exhibit stronger antioxidant activity than larger polypeptides due to better ability to cross the intestinal barrier and have an easier interaction with free radicals (Chi, Wang, Wang, Zhang, & Deng, 2015; Hsu, 2010). Our results are in accordance with Ranathunga et al. (2006) and Jang et al. (2016) who also found higher antioxidant activity by low molecular weight peptides. A few amino acids have been reported to be crucial to the antioxidant activity of peptides. The antioxidant activity of peptides containing hydrophobic amino acids contributes to an increase in their lipid solubility, which facilitates access to hydrophobic radical species. Moreover, hydrophobic amino acids are able to promote entry of the peptide into target organs through hydrophobic association, which is achieved favorably due to antioxidant properties (He et al., 2013; Sarmadi & Ismail, 2010). In this study, hydrophobic amino acid residues Ile, Pro, and Leu were present in the purified fraction PAP2, which could explain its high antioxidant activity. Wang, Camp, and Ehlenfeldt (2012) reported that Ser and Cys can play an essential role in antioxidant effect due to the presence of hydroxyl and sulfhydryl groups, and aromatic amino acids can donate protons to electron‐deficient radicals while keeping their own stability through resonating structure (Rajapakse et al., 2005). Memarpoor‐Yazdi, Mahaki, and Zare‐Zardini (2013) indicated that the presence of basic amino acids such as Arg within the peptide sequence was effective on its metal ion chelating activity. Therefore, based on the results obtained in this study, we assume that Leu, Ile, Ser, Tyr, and Arg in PAP2 played an important role in enabling antioxidant peptides to function as potent radical scavengers.

## 4 CONCLUSION

In the present study, the proteins of perilla seed by‐products (PSM) were hydrolyzed by trypsin to obtain antioxidant peptides. The PSM protein hydrolysate was purified through ultrafiltration and reverse‐phase HPLC (preparative and analytical). The amino acid sequence of the purified antioxidant peptide (PAP1) derived from fraction IV could not be confirmed. For PAP2 from fraction V, the amino acid sequence was identified as Ile‐Ser‐Pro‐Arg‐Ile‐Leu‐Ser‐Tyr‐Asn‐Leu‐Arg, with a molecular weight of 1,330.77 Da. The purified peptides exhibited good antioxidant activities, such as reducing power, DPPH radical scavenging activity, and ABTS radical scavenging activity. The high activity of purified antioxidant peptides was due to their low molecular weight (<3 kDa) and the presence of specific amino acids including Leu, Ile, Pro, and Ser. These results suggest that hydrolysates from PSM can be used as natural antioxidants. However, further studies on antioxidant activities in vivo are needed.

## CONFLICT OF INTEREST

The authors declare that they do not have any conflict of interest.

## ETHICAL APPROVAL

This study does not involve any human or animal testing.
